# Mutations in α-Tubulin Cause Abnormal Neuronal Migration in Mice and Lissencephaly in Humans

**DOI:** 10.1016/j.cell.2006.12.017

**Published:** 2007-01-12

**Authors:** David A. Keays, Guoling Tian, Karine Poirier, Guo-Jen Huang, Christian Siebold, James Cleak, Peter L. Oliver, Martin Fray, Robert J. Harvey, Zoltán Molnár, Maria C. Piñon, Neil Dear, William Valdar, Steve D.M. Brown, Kay E. Davies, J. Nicholas P. Rawlins, Nicholas J. Cowan, Patrick Nolan, Jamel Chelly, Jonathan Flint

**Affiliations:** 1 Wellcome Trust Centre for Human Genetics, University of Oxford, Oxford, OX3 7BN, UK; 2 Department of Biochemistry, New York University Medical Center, New York, NY10016, USA; 3 MRC Functional Genetics Unit, South Parks Road, Oxford, OX1 3QX, UK; 4 MRC Mammalian Genetics Unit, Harwell, Didcot, OX11 0RD, Oxfordshire, UK; 5 Department of Pharmacology, The School of Pharmacy, 29-39 Brunswick Square, London, WC1N 1AX, UK; 6 Department of Physiology, Anatomy and Genetics, University of Oxford, South Parks Road, Oxford, OX1 3QX, UK; 7 Department of Experimental Psychology, University of Oxford, South Parks Road, Oxford, OX1 3UD, UK; 8 Institut Cochin, INSERM Unité 567, CNRS UMR 8104, Université René Descartes – Paris 5, Faculté de Médecine René Descartes, Paris, F-75014, France

## Abstract

The development of the mammalian brain is dependent on extensive neuronal migration. Mutations in mice and humans that affect neuronal migration result in abnormal lamination of brain structures with associated behavioral deficits. Here, we report the identification of a hyperactive N-ethyl-N-nitrosourea (ENU)-induced mouse mutant with abnormalities in the laminar architecture of the hippocampus and cortex, accompanied by impaired neuronal migration. We show that the causative mutation lies in the guanosine triphosphate (GTP) binding pocket of α-1 tubulin (*Tuba1*) and affects tubulin heterodimer formation. Phenotypic similarity with existing mouse models of lissencephaly led us to screen a cohort of patients with developmental brain anomalies. We identified two patients with de novo mutations in *TUBA3*, the human homolog of *Tuba1*. This study demonstrates the utility of ENU mutagenesis in the mouse as a means to discover the basis of human neurodevelopmental disorders.

## Introduction

The development of the mammalian brain depends on extensive neuronal migration resulting in the formation of a number of highly laminar structures, most notably the cortex and hippocampus. This process involves a large number of neurons that originate in the proliferative ventricular zones and migrate radially to their final locations past previously formed neurons (Gupta et al., 2002). Genetic studies in mice and humans have identified some of the molecular determinants of neuronal migration: mutations in doublecortin (*DCX*) (des Portes et al., 1998, Gleeson et al., 1998) and *LIS1* (Reiner et al., 1993) have been shown to impair migration and cause type 1 lissencephaly in humans, a disease characterized by a four-layered cortex with an absence or diminution of gyri and sulci (Dobyns and Truwit, 1995). In females, mutations in *DCX* can also cause subcortical laminar heterotopia (SCLH), in which an additional layer of misplaced heterotopic neurons is found in the white matter. Perturbations of the laminar architecture of the cortex also result from mutations in the very low density lipoprotein receptor (*VLDLR)* (Boycott et al., 2005) and the extracellular matrix protein *Reelin* (Hong et al., 2000). Together, mutations in *DCX*, *LIS1*, *VLDLR*, and *reelin* are responsible for ∼70% of the cases of type 1 lissencephaly. The pathogenic mutations responsible for the remaining cases are unknown (Leventer, 2005).

Impaired neuronal migration affects behavior as well as brain structure. Humans with lissencephaly are mentally retarded and frequently suffer from epilepsy (Francis et al., 2006). Similarly, the *reeler* mouse mutant and mice with loss of function mutations in *Dcx* and *Lis1* exhibit varying degrees of cognitive dysfunction (Corbo et al., 2002, Paylor et al., 1999, Qiu et al., 2005). Mice with mutations that cause behavioral abnormalities are currently being identified as part of a large-scale mutagenesis screen in the UK (Nolan et al., 2000). In this dominant screen, N-ethyl-N-nitrosourea (ENU) is injected into the peritoneum of BALB/cAnN male mice and causes random mutations in spermatogonia. Mutagenized males are mated with C3H/HeH females and their offspring assessed for variation in locomotor activity. Here we describe a hyperactive ENU-induced mouse mutant with cortical and hippocampal abnormalities due to impaired neuronal migration. We show that mutations in the human homolog of this gene cause cortical brain malformations, including lissencephaly.

## Results

### Identification and Mapping of a Novel Behavioral Mutant

**Figure 1:**
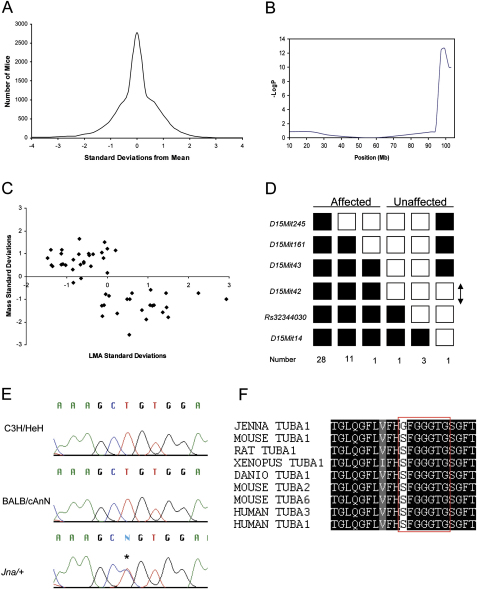
Behavioral Screen and Genetic Mapping (A) Results of the locomotor activity screen showing the distribution of mice as a function of the number of standard deviations from the mean. (B) Genetic mapping results for total beam breaks on chromosome 15 when analyzed with QTL cartographer employing a linear regression. Significant linkage was obtained to distal chromosome 15 (LogP = 12.6). (C) Correlation between weight and locomotor behavior. Mice fall into two groups: (1) low activity mice with a larger mass and (2) hyperactive mice with a smaller mass. (D) Fine mapping for the *Jna* pedigree. Black boxes represent heterozygosity for the BALB/cAnN allele and the white boxes homozygotes C3H/HeH. The number of progeny inheriting each haplotype is listed on the bottom line. Haplotype analysis indicated that the mutation falls within a 1.3 Mb region between *D15Mit43* and *rs32344030*, shown with an arrow. (E) Sequencing traces from exon 4 in *Tuba1* from C3H/HeH, BALB/cAnN, and an affected *Jna/+* mouse. The mutation, highlighted with a star, is a T to C transition. (F) Alignment of α-tubulin isotypes from various species. The mutation in the *Jna/+* mouse substitutes a serine with a glycine residue that is conserved in all α-tubulins. The boxed residues are known to interact directly with GTP.

We screened 9216 mice from the Harwell ENU mutagenesis program. The total distance each mouse travelled in 35 min was assessed. Mice with levels of activity more than three standard deviations outside the mean were retested 7 days later (n = 87) (Figure 1A). We excluded mice with abnormal vestibular, skeletal, or neuromuscular phenotypes that might also influence locomotor behavior. Ten outliers were subject to heritability testing by backcrossing to C3H/HeH. A single line that we named Jenna (Jna) was identified with a semidominant hyperactive phenotype.

We mapped variation in locomotor activity to the distal end of chromosome 15 by treating the phenotype quantitatively and applying standard methods for quantitative trait locus (QTL) mapping (Zeng, 1994). To generate sufficient animals to map the mutation, we employed in vitro fertilization, raising a total of 89 animals on a C3H/HeH background. We attempted to intercross hyperactive animals but failed to generate any pregnancies.

We identified a QTL on chromosome 15 with a logP (negative logarithm of the P value, base 10) of 12.6. This was the only locus that exceeded the genome-wide 5% significance threshold of logP 4.1 (Churchill and Doerge, 1994). No other loci were detected with a logP greater than 2.0. The presence of a relatively large QTL segregating on chromosome 15 suggested that we had mapped the causative mutation (Figure 1B), but we could not exclude the possibility that the genetic effect arose from a naturally occurring polymorphism between BALB/cAnN and C3H/HeH.

We reasoned that the presence of pleiotropy would distinguish the effect of the ENU-induced mutation from a naturally occurring polymorphism. Unlike the latter, engineered and induced mutations with an effect on behavior frequently display additional phenotypic abnormalities. We found that mice from the mapping population could be divided into two groups by variation in weight and activity, the smaller mice exhibiting higher levels of activity (Figure 1C). We then fine-mapped the mutation to a 1.3 Mb region between *D15Mit43* and a single nucleotide polymorphism *rs32344030* (Figure 1D). This region contains 41 annotated genes (http://www.ensembl.org).

The coding and exon-flanking sequences of all 41 genes was sequenced in affected animals (n = 4), a wild-type control, and the two mapping strains, C3H/HeH and BALB/cAnN. All the variants identified were present in the two mapping strains, except for a T to C transition in exon 4 of α-1 tubulin (*Tuba1*) (Figure 1E). We sequenced exon 4 of α-1 tubulin from the inbred strains AKR/J, A/J, C57BL6/J, RIII, DBA/2J, IS, FVB, and 129 and did not observe this polymorphism.We extracted whole brain mRNA from *Jna/+* animals, sequenced the cDNA, and confirmed the presence of the mutation in the brain *Tuba1* transcript. The mutation causes an amino acid change from serine to glycine at residue 140 (S140G). This amino acid is located in the T4 loop of Tuba1, forming part of the N-site, a highly conserved glycine-rich motif that binds GTP (Lowe et al., 2001) (Figures 1F and 7B). GTP bound at the N site does not undergo hydrolysis and is thought to act as a structural cofactor stabilizing the α/β heterodimer (Spiegelman et al., 1977).

### The S140G Mutation Reduces GTP Binding and Native Heterodimer Formation

**Figure 2:**
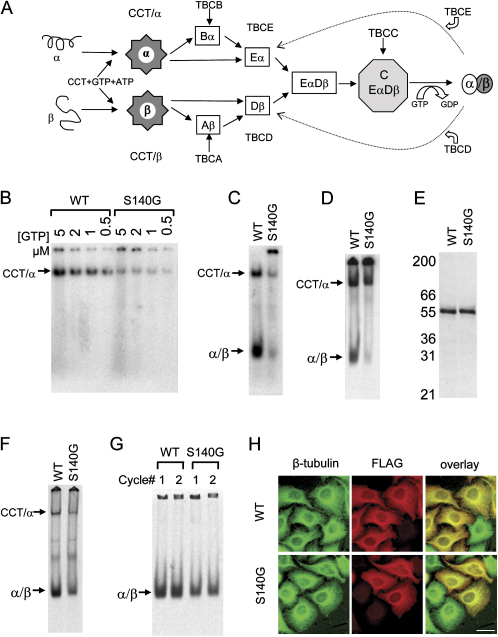
Tuba1 S140G Has a Reduced Ability to Incorporate GTP, Resulting in a Marked Decline in the Efficiency of Tubulin Heterodimer Formation (A) The tubulin heterodimer assembly pathway. Newly synthesized α/β-tubulin polypeptides undergo one or more rounds of ATP-dependent interaction with cytosolic chaperonin (CCT). Quasi-native intermediates formed as a result of this interaction are then captured and stabilized by a series of tubulin-specific chaperones termed TBCA–TBCE. α-tubulin intermediates interact with TBCB and TBCE, while β-tubulin intermediates interact with TBCA and TBCD. TBCEα (Eα) and TBCDβ (Dβ) together form a supercomplex. Entry of TBCC into this supercomplex triggers GTP hydrolysis by β-tubulin; this acts as a switch for the discharge of the newly assembled α/β heterodimer. This is based on Tian et al. (2006). (B) Incorporation of labeled GTP into CCT bound folding intermediates. Reaction products, analyzed by nondenaturing gel electrophoresis, show an approximately 5-fold reduction in GTP bound intermediates (CCT/α) in the case of the S140G mutant. (C) Incorporation of labeled GTP in fully reconstituted in vitro tubulin-folding reactions containing CCT, ATP, GTP, TBCB, TBCC, TBCD, and TBCE; native brain tubulin; and equal amounts of wild-type or S140G mutant α-tubulin. Products analyzed by nondenaturing gel electrophoresis show a reduction in the level of native α/β-tubulin heterodimers. (D) Incorporation of ^35^S-labeled wild-type or mutant (S140G) Tuba1 in fully reconstituted in vitro reactions confirm a reduction in the production of native α/β-tubulin heterodimers in the case of the S140G mutant. (E and F) In vitro transcription and translation in rabbit reticulocyte lysate in the presence of ^35^S-methionine. Products analyzed by SDS (E) or nondenaturing gel electrophoresis (F) demonstrate that the mutation does not affect translation of the protein (E) in contrast to the diminished yield of heterodimers in (F). In (E) molecular mass markers (in kDa) are shown on the left. (G) Microtubule polymerization/depolymerization. Cycling of ^35^S-labeled, in vitro translated wild-type and mutant (S140G) tubulin with native bovine brain microtubules demonstrates that wild-type and mutant heterodimers are equally capable of incorporation into microtubules. (H) The S140G mutant α-1 tubulin incorporates into microtubules in vivo. C-terminally FLAG-tagged wild-type or mutant Tuba1 were transfected into HeLa cells and the microtubule network visualized by staining with a polyclonal anti-β-tubulin antibody (shown in green) and a monoclonal anti-FLAG antibody (shown in red). Calibration bar shows 10 μm. Arrows in panels (D), (E), (F), and G denote the location of the α-tubulin/CCT binary complex (CCT/α) or the native tubulin heterodimer (α/β).

We hypothesized that the S140G mutation might affect the ability of Tuba1 to bind GTP at the N site. We therefore conducted in vitro folding assays containing α-^32^P-GTP. Equal amounts of urea-unfolded wild-type or mutant Tuba1 probes, quantitated by Coomassie blue staining, were introduced by sudden dilution into a buffer containing ATP, radio-labeled GTP, and cytosolic chaperonin (CCT). CCT is the first chaperone required in a cascade of interacting proteins that act in concert to facilitate the formation of native tubulin heterodimers (Figure 2A) (Lewis et al., 1996). CCT undergoes multiple rounds of ATP-dependent interaction with unfolded α-tubulin polypeptides, forming chaperonin bound quasi-native tubulin folding intermediates that contain a GTP binding pocket (Tian et al., 1995).

We found that the S140G mutation decreased the ability of CCT bound quasi-native α-tubulin folding intermediates to incorporate GTP by approximately 5-fold (Figure 2B). We then assessed what effect this would have on native tubulin heterodimer formation. To do this, we repeated the assay, including the additional components required for de novo heterodimer formation. We found that the yield of labeled heterodimers was significantly less in reactions carried out with the mutant protein compared to the wild-type protein (Figure 2C).

To show that the reduced incorporation of GTP in mutant heterodimers is reflected in a reduced yield of native dimerized protein, we repeated our in vitro folding experiments using mutant and wild-type unfolded ^35^S-methionine labeled Tuba1 probes. Consistent with the data obtained in our GTP labeling experiments, we found that the S140G mutation reduced the efficiency of de novo heterodimer formation under conditions where equal amounts of ^35^S-labeled protein were analyzed (Figure 2D). These data are not artifacts of in vitro reconstitution, because we observed the same reduced yield of tubulin heterodimers in parallel experiments in which the identical cloned sequences used for the generation of probes for in vitro folding experiments were translated in a cell free system from rabbit reticulocytes (Figures 2E and 2F). We then investigated whether the heterodimers produced in these in vitro experiments were able to polymerize. The products of the reactions shown in Figure 2D were mixed with depolymerized brain microtubules and taken through two successive cycles of polymerization/depolymerization. At the end of each cycle, an aliquot of the depolymerized material was removed and analyzed. While heterodimer formation was reduced in all our assays carried out with the S140G mutant, the ability of mutant heterodimers to cocycle with native tubulin in vitro was not affected (Figure 2G).

Consistent with these data we found that a FLAG-tagged mutant Tuba1 incorporated into the normal interphase microtubule network upon overexperession in cultured cells (Figure 2H) and that these microtubules behaved in the same manner as their wild-type counterparts in a regrowth assay following depolymerization with nocodazole (Figure S1). These experiments demonstrate the ability of the S140G tubulin, once folded, to assemble into heterodimers that can copolymerize into dynamic microtubules in vivo. We conclude that the S140G mutation results in a diminished efficiency of polymerization-competent tubulin heterodimer formation as a result of a compromised GTP binding pocket. Our data indicate that the S140G substitution is a partial loss-of-function mutation, suggesting that the phenotype in *Jna/+* mice is due to haploinsufficiency.

### Genetic Rescue of the Hyperactive Phenotype in *Jna/+* Mice

We tested whether delivery of additional copies of α-1 tubulin by BAC transgenesis would rescue the phenotype in *Jna/+* mice. We selected a BAC clone (RP23-3124H4) that contained the *Tuba1* gene for this experiment. Purified BAC DNA was injected into the pronucleus of C3H/HeH embryos, and these embryos transferred into recipient females. Four transgenic lines were identified that carried the BAC (H2, H8, H17, and H41). We established germline transmission in two of these (H8 and H41). Quantitative PCR analysis of the transgene indicated that four copies of the clone had been incorporated into the H41 line and a single copy into the H8 line (data not shown). Behavioral analysis of the H41 line revealed no significant differences in measures of activity with control animals (F[1,16] < 1; P > 0.5), demonstrating that delivery of additional copies of *Tuba1*, by itself, has no effect on locomotor behavior (Figure 5A). The H41 line was selected for rescue and was crossed with *Jna/+* mice. There was a significant reduction in the locomotor behavior of transgenic animals with the S140G mutation (*Jna*/+/BAC) in comparison to nontransgenic mutants (F[1,14] = 18.7; P < 0.005) and no significant difference from controls (F[1,16] = 1.2; P > 0.1) (Figure 5A).

### The S140G Mutation Results in Abnormal Hippocampal and Cortical Morphology in *Jna/+* Mice

**Figure 3:**
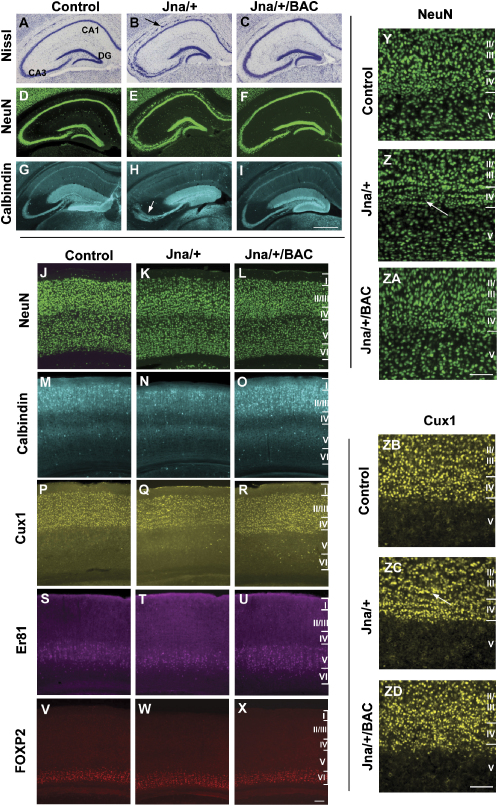
Abnormal Hippocampal and Cortical Morphology in *Jna/+* Mice (A–I) Coronal sections of the hippocampus from littermate controls, heterozygote (*Jna*/+) and rescued (*Jna*/+/BAC) animals aged 8 weeks when stained with cresyl violet (A–C), NeuN (D–F), and calbindin (G–I). These stains reveal a fractured pyramidal cell layer (shown with an arrow) that is most severe in the CA3, fewer calbindin-positive pyramidal neurons in the CA1 region, and a disorganized mossy fiber tract (arrowed). (J–ZD) Coronal sections of the visual cortex when stained with anti-sera for NeuN (J–L, Y–ZA), calbindin (M–O), Cux-1 (P–R, ZB–ZD), Er81 (S–U), and FOXP2 (V–X) from mice aged 8 weeks. These stains showed that the laminar structure of the cortex is preserved; however, on closer examination of NeuN (Y–ZA) and Cux-1 (ZB–ZD) staining, wave-like perturbations in layers II/III and IV (arrowed) can be seen. The calibration bar for the hippocampus shows 500 μm. The calibration bars for the cortex show 200 μm.

We undertook a complete histological examination of *Jna/+* mice (n = 4). Haematoxylin and eosin staining revealed no gross anatomical defects in somatic organs or tissues (data not shown). We focused on the neuroanatomical features of the *Jna/+* mice. Brains from *Jna/+* mice were smaller than wild-type (∼85%), but this is probably because mutant animals weigh ∼30% less than littermate controls. Examination of coronal sections of *Jna/+* mice (n = 6), littermate controls (n = 6), and rescued brains (n = 6) from animals aged 8 weeks revealed abnormal lamination of pyramidal cells in the hippocampus of mutant animals (Figures 3A–3I). Staining with cresyl violet and with the neuronal marker NeuN showed hippocampal disorganization with an additional layer of pyramidal cells in the stratum oriens that extended throughout the pyramidal cell subfields into the subiculum, which was most marked in the CA3 region where the neurons were loosely packed. The abnormality was also apparent in mutant animals aged 8 months (n = 3) (data not shown). Staining with calbindin revealed fewer calbindin-positive pyramidal neurons in the CA1 region and a disorganized mossy fiber tract extending from the dentate gyrus to CA3. No gross morphological abnormalities were apparent in the dentate gyrus of *Jna/+* mice, the hilus appearing intact. There were no detectable abnormalities in the hippocampal morphology of *Jna*/+/BAC mice (Figures 3C, 3F, and 3I).

We looked for laminar abnormalities in the cortex, staining with antisera for NeuN, Cux-1 (a layer II/III/IV marker), calbindin (a layer II/III and V marker), Er81 (a layer V marker), FOXP2 (a layer VI marker) (Figure 3 J-X), Brn-1 (expressed predominantly in late cortical plate neurons, Figure S4), and cresyl violet (Figure S2). These experiments showed that the laminar cytoarchitecture of the cortex was intact in *Jna/+* mice, but closer examination of NeuN, Cux-1, and Nissl stains revealed wave-like perturbations in layer IV (Figure 3, Y-ZA and ZB-ZD; Figure S2). While layer IV still consisted of the characteristic granular cells that were of similar size and shape to those observed in littermate controls, they were organized into three to four cellular fronts, each containing a single row of cells. This perturbation was evident in the visual, auditory, and somatosensory cortices but was not observed in the motor or retrosplenial cortices (Figure S2). It was observed primarily in layer IV but extended into layers II/III in the posterior and dorsal medial regions of the cortex as revealed by Cux-1 staining. *Jna*/+/BAC mice were indistinguishable from wild-type mice for all stains (Figure 3, L, O, R, U, and X). Analysis of Golgi- stained sections revealed no significant differences in the percentage of misorientated (θ > 8°) or inverted (θ > 90°) apical dendrites in the visual, somatosensory, or motor cortices between littermate controls (n = 3) and *Jna/+* mice (n = 3) (Demyanenko et al., 2004) (Figure S3). No anatomical abnormalities were seen in the cerebellum or amygdala (Figure S4).

### Defective Neuronal Migration in *Jna/+* Mice

**Figure 4:**
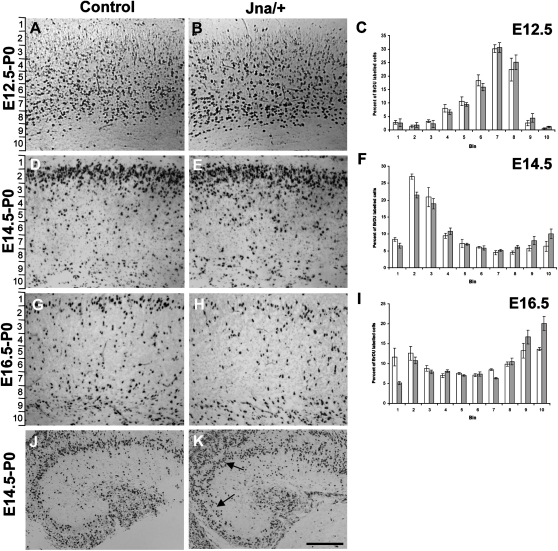
Abnormal Neuronal Migration in *Jna/+* Mice (A–I) Staining for BrdU in the cortex and hippocampus of littermate controls and mutant pups harvested at P0, after injection of BrdU at E12.5 (A and B), E14.5 (D and E) and E16.5 (G and H). The cortex was divided into ten equal bins (shown on the left), extending from the intermediate zone to the molecular layer and BrdU-positive cells counted blind to the genotype. Panels (C), (F), and (I) show the mean percentage of BrdU-labeled cells in bins 1 to 10 for wild-type littermates (white) and *Jna/+* mutants (gray). Error bars show the SEM. Test of the interaction between genotype and the distribution of cells across bins were as follows: E12.5 (F[9,189] < 1; P > 0.05); E14.5 (F[9,198] = 4.75; P < 0.0001), and E16.5 (F[9,270] = 13.3; P < 0.0001). (J and K) BrdU-positive cells in the hippocampus of a littermate control (J) and *Jna/+* mouse (K) following injection of BrdU at E14.5. *Jna/+* mice show disorganization of BrdU-positive cells in Ammon's horn, affecting both CA1 and CA3 regions (arrowed). Scale bar shows 200 μm (J and K).

We considered whether abnormal radial migration might be responsible for the unusual hippocampal and cortical architecture observed in the *Jna/+* mice. We injected pregnant females with BrdU at three time points (E12.5, E14.5, and E16.5) and counted labeled cells at the day of birth (P0), dividing the cortex into ten equal bins extending from the intermediate zone to the cortical surface (Figures 4A–4C). We tested for an interaction between genotype and the mean percentage of cells in each bin. There was no significant difference between littermate controls and *Jna/+* mutants when BrdU was injected at E12.5 (F[9,189] < 1; P > 0.05), however there was a highly significant difference when injected at E14.5 (F[9,198] = 4.75; P < 0.0001), and at E16.5 (F[9,270] = 13.3; P < 0.0001). At these time points, a higher percentage of BrdU-positive cells are observed in bins 1, 2, and 3 in wild-type littermates. These results are consistent with our anatomical observations that showed perturbations in layers II/III and IV of the cortex, as the majority of cells that populate these layers are born between E14.5 and E16.5 (Takahashi et al., 1999).

Examination of hippocampal sections following injection of BrdU at E14.5, a time at which most pyramidal neurons are born (Angevine, 1965), revealed disorganization in Ammon's horn affecting both CA1 and CA3 regions (Figures 4D and 4E). Several layers of BrdU-positive cells could be seen, lacking structure when compared to wild-type littermates. The dentate gyrus, unlike the phenotype in the adult, also appeared chaotic, suggesting a delay in its development. This result, together with our analysis of BrdU staining in the cortex, is consistent with impaired radial migration in *Jna/+* mice.

### *Jna/+* Mice Show Impaired Spatial Working Memory, Reduced Anxiety, and Abnormal Nesting Consistent with a Hippocampal Deficit

Given the lamination defect in the hippocampus, we tested whether hippocampal-dependent behaviors are altered in *Jna/+* mice (Deacon et al., 2002). A number of behaviors are known that require an intact hippocampus. Effects on memory are well documented, but lesions also result in increased activity, reduced anxiety, and disruption of a number of species-typical behaviors (Deacon et al., 2002). We were unable to transfer the S140G mutation into a C57BL/6 background for behavioral testing, so all phenotyping was conducted on a C3H/HeH background.

**Figure 5:**
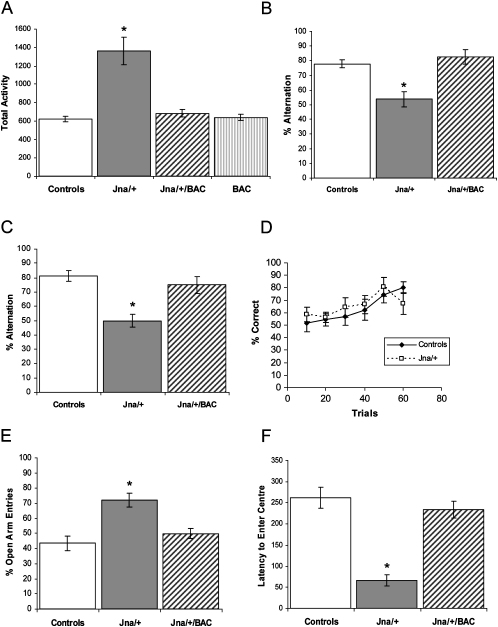
Abnormal Behavior in *Jna/+* Mice (A) Total beam breaks, a measure of locomotor activity, for *Jna/+* mice, wild-type littermates, mutant animals with the transgene (*Jna*/+/BAC), and the H41 transgenic line (BAC). Addition of the transgene alone has no effect on locomotor activity (F[1,16] < 1; P > 0.5) but rescues the hyperactive phenotype in *Jna/+* mice. (B and C) In comparison to wild-type littermates, *Jna/+* mice show impaired hippocampal-dependent working memory when assessed by discrete trial spontaneous (F[1,12] = 34.1; P < 0.0001) and rewarded alternation (F[1,12] = 29.84; P < 0.0005). (D) *Jna/+* mice exhibit no deficits when assessed on a nonspatial reference memory task that relies on tactile discrimination. (E and F) *Jna/+* mice show a low anxiety phenotype, entering the open arms of the elevated plus maze more often (F[1,15] = 17.7; P < 0.001) and the center of the open field sooner (F[1,16] = 46.7; P < 0.0001) than littermate controls. Error bars show the SEM.

We assessed the *Jna/+* mice for spontaneous alternation in a T-maze, a working memory task that does not require vision (Buhot et al., 2001, Farr et al., 2002). *Jna/+* mice performed significantly worse than littermate controls (F[1,12] = 34.1; P < 0.0001) (Figure 5A), alternating just above chance. Similar results were obtained when working memory was assessed with rewarded alternation, *Jna/+* mice again exhibiting a significant reduction in the percentage of alternation in comparison to controls (F[1,12] = 29.84; P < 0.0005) (Figure 5B). We considered whether the abnormality of spatial working memory could be attributed to deficits in memory acquisition that do not depend on the hippocampus. We assessed *Jna/+* mice on a hippocampal-independent reference memory acquisition task. This experiment utilized a T-maze with a number of different floor inserts (carpet, sandpaper, thin wire mesh, or wire bars). Mice were trained to associate a food reward with a particular insert in a counter-balanced protocol. Both mutant and control mice were able to learn this task (Figure 5C) (F < 1; P > 0.5).

To assess hippocampal-dependent anxiety in the *Jna/+* mice, we used an elevated plus maze and open field. *Jna/+* mice preferred the open arms of the elevated plus maze, indicating that in addition to being hyperactive, they are less fearful than sibling controls (F[1,15] = 17.7; P < 0.001) (Figure 5D). Mutants also showed a reduced latency to enter the center of the open field (F[1,16] = 46.7; P < 0.0001) (Figure 5E). We also tested nesting, a species-typical behavior that is sensitive to hippocampal lesions (Deacon et al., 2002). We found that mutant animals failed to construct proper nests (Z = −3.4; P = 0.001) (data not shown). The poor performance of the *Jna/+* mice on this task is not due to a general deficit in motor coordination, as they perform well on the accelerating rotarod and on the static rods test (data not shown). Control and rescued animals showed no statistically significant differences for spontaneous alternation (F[1,12] < 1; P > 0.1), rewarded alternation, the percentage of open arm entries (F[1,16] = 1.1; P > 0.1), latency to enter the center of the open field (F[1,16] < 1; P > 0.1) and nesting score (Z = −0.3; P > 0.5).

### Mutations in α-Tubulin Cause Lissencephaly in Humans

**Figure 6:**
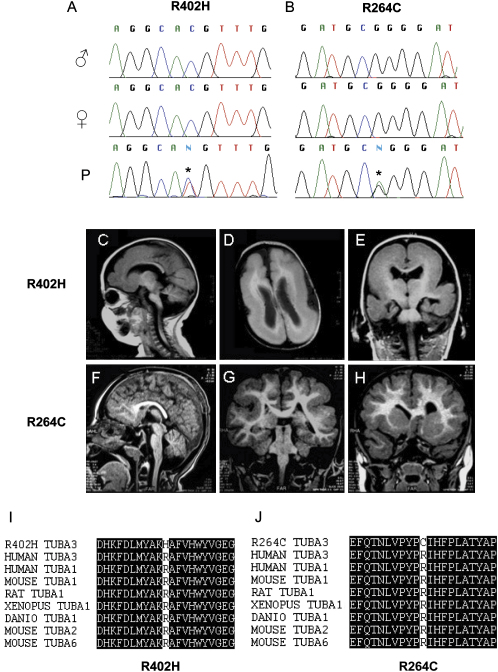
Mutations in *TUBA3* Cause Lissencephaly in Humans Screening of lissencephalic patients without mutations in *DCX* or *LIS1* identified two individuals with spontaneous pathogenic mutations in *TUBA3*. (A and B) The sequencing traces for the father (♂), mother (♀), and affected patient (P) for these two individuals. In the first, a C to T substitution mutates an arginine residue to histidine (R402H), and, in the second, a G to A substitution mutates another arginine to cysteine (R264C). (C–E) The R402H patient shows severe lissencephaly. (F–H) The second individual exhibits less severe cortical abnormalities. (I and J) Both these residues are highly conserved in different species and isotypes of α-tubulin.

The similarity of the anatomical and behavioral phenotypes of the *Jna/+* mice to the *Dcx*, *Lis1*, and *reeler* mouse mutants led us to consider whether mutations in the human homolog of *Tuba1* might cause lissencephaly in humans. We searched for mutations in the human homolog of α-1 tubulin, *TUBA3,* in a cohort of patients in whom MRI investigation had shown the presence of cortical dysgeneses. Previous molecular studies had excluded mutations in *DCX* and *LIS1* genes in these patients (n = 40). We identified two patients with pathogenic mutations in *TUBA3.* One patient with severe epilepsy, mental retardation, and motor deficits, shows classic lissencephaly with a thick disorganized cortex (Figure 6C–6E). The causative mutation, a C to T substitution, mutates a highly conserved arginine residue to a histidine (R402H) at the beginning of the H11-H12 loop near the interface of *TUBA3* with β-tubulin (Figure 6A, 6I, and 7C). The second patient exhibits less severe cortical abnormalities, with pachygyria at the temporal level and around the Rolandic sulcus, accompanied by abnormal organization of the hippocampus (Figure 6F–6H). The mutation, a G to A substitution, mutates an arginine to cysteine (R264C) located at the surface of α-tubulin in a loop between H8 and S7, again in a highly conserved region of the protein (Figure 6B, 6J, and 7D). Both patients show agenesis of the splenium of the corpus callosum, abnormalities of the inferior vermis, and hypoplasia of the brain stem.

**Figure 7:**
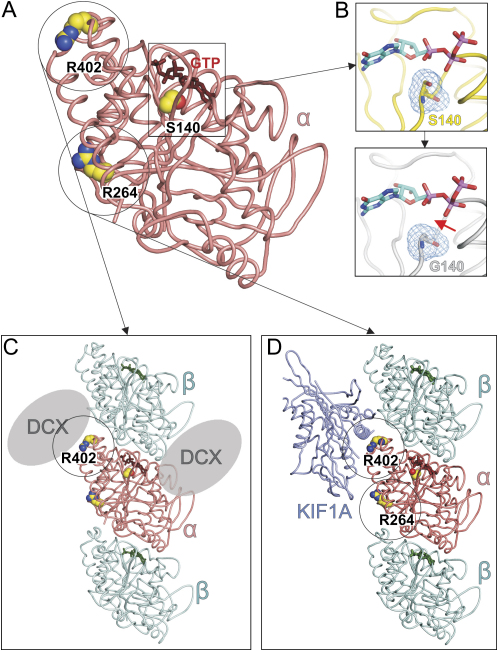
Mapping of the Mutations onto the α-Tubulin Structure (A) The structure of α-tubulin (pdb: 1JFF). The three mutations are highlighted as spheres in atomic coloring (nitrogen: blue; oxygen: red; carbon: yellow). The bound GTP molecule is shown as red sticks. (B) Close-up view of the GTP binding site of α-tubulin. Serine 140, located on the T4 loop, is depicted as a stick presentation in atomic coloring according to (A) with its solvent accessible surface shown as blue wire. The second panel shows a model of the S140G mutation, the red arrow indicating extra space generated by the mutation and potentially responsible for disrupting the interaction with GTP. (C) Schematic presentation of the tubulin:doublecortin complex based on a structural model from Moores (Moores et al., 2004). The position of arginine 402 (R402), located at the beginning of the H11-H12 loop, is highlighted with a circle (α-tubulin: salmon; β-tubulin: cyan; doublecortin: gray ellipse). (D) Cartoon presentation of the complex between tubulin and the kinesin KIF1A (pdb: 2HXF). KIF1A is colored in slate. The positions of arginine 264 (R264), located at the surface of the molecule at the loop between H8 and S7, and of argininine 402 (R402), are highlighted by circles.

Having excluded nonpaternity by typing informative polymorphic markers in parents and offspring, we screened the TUBA3 gene for sequence variants in the patients' parents (all phenotypically normal) and 360 controls. We found no coding sequence variants in the parents or controls, demonstrating their de novo occurrence and their absence in the normal population. The frequency of mutations is significantly different between cases and controls (P = 0.01). Screening of TUBA3 in a second cohort of patients with cortical anomalies identified a further six individuals with de novo mutations in TUBA3. Mutated residues again include R402 and R264 (unpublished data). Our results indicate that mutations in TUBA3 cause human cortical migration disorders.

## Discussion

We report the cloning of an ENU-induced mutation that underlies abnormal hippocampal-dependent behavior in the mouse. The mutation, an S140G substitution in Tuba1, impairs the ability of the protein to bind GTP and to form native heterodimers with β-tubulin. Heterodimers that do form are able to polymerize and are incorporated into the microtubule network of cultured cells. The mutation results in abnormal neuronal migration in vivo; perturbations in layers II/III and IV of the visual, auditory, and somatosensory cortices; and a fractured pyramidal cell layer in the hippocampus.

### Tuba1, Microtubules, and Neuronal Migration

We believe that the S140G mutation asserts its effect by interfering with microtubule function, thereby impairing neuronal migration. First, we have shown that the S140G mutation affects GTP binding and impairs tubulin heterodimer formation. Studies in yeast have shown that mutations in tubulin that influence heterodimer formation can result in microtubules with aberrant properties (Vega et al., 1998). Second, correct microtubule function is important for key processes involved in neuronal migration, including neurite extension and nucleokinesis (Kappeler et al., 2006, Phillips et al., 2004). Third, the expression of *Tuba1* coincides with the time of neuronal birth at E9.5 and is maintained along defined migratory pathways in the neocortex (Gloster et al., 1999). Finally, we have employed BrdU in birthdating studies to demonstrate an in vivo neuronal migration deficit in *Jna/+* mice.

Our data are consistent with a model in which the abnormal phenotype observed in the *Jna/+* mice arises from a reduction in the total amount of functional heterodimers. A consequence of reduced heterodimers might be a reduction of incorporated S140G tubulin into microtubules, which may in turn affect polymerization dynamics. We have not observed a reduction in the incorporation of S140G tubulin in our cellular assay, although this may be the consequence of overexpression by the cytomegalovirus (CMV) promoter in an epithelial cell line. An alternative hypothesis is that an imbalance in the relative levels of expression of α/β-tubulin could be a contributing factor (Vega et al., 1998). A third possibility is that the S140G mutation alters the stability of microtubules. Microtubule destabilization is believed to be the mechanism whereby mutations in the *Aspergillus nidulans* homolog of α-tubulin suppress nuclear migration mutations (Willins et al., 1995).

### TUBA3 Mutations Cause Lissencephaly in Humans

Mutations in *TUBA3*, the human homolog of α-1 tubulin, are present in two patients with type 1 lissencephaly and pachygyria. Structural studies reveal that neither of these mutations (R402H and R264C) lie in the GTP binding domain of TUBA3, suggesting they do not influence GTP binding. While it is possible that these mutations affect the folding of tubulin heterodimers, they may also influence interactions with proteins that bind microtubules, particularly the R402H mutation. The R402H mutation is located in a loop of α-1 tubulin, that forms an interface with doublecortin (Moores et al., 2004) (Figure 7C), and the kinesin KIF1A (Kikkawa and Hirokawa, 2006) (Figure 7D). It remains to be determined whether these interactions are affected by the R402H mutation.

Our data demonstrate the utility of ENU mutagenesis in the mouse as a means to discover the basis of human neurodevelopmental disorders. *TUBA3* should now be screened in individuals with cortical disorders, especially where cortical dysgenesis is associated with agenesis of the corpus callosum, abnormalities of the inferior vermis, and hypoplasia of the brain stem.

## Experimental Procedures

### Mice

Mice were maintained on a 12:12 light:dark cycle at a temperature of 22 ± 1°C with a humidity of 60%–70%. Males and females were housed separately, and where possible five mice were placed in each cage. Animals had ad libitum access to food except during appetitively motivated tasks, where mice were maintained at 85% of their free-feeding weight. Experiments were performed in accordance with the UK Animals (Scientific Procedures) Act 1986.

### Behavioral Screen

F1 mice were screened for abnormal locomotor activity at 6–7 weeks of age. Activity was assessed in a novel environment (a rat cage with fresh sawdust under fluorescent lights) for 35 min. A beam-splitting device (Benwick Electronics) employing infrared technology recorded the number of beam breaks and cage transitions in 5 min allocations. Values in the first 5 min were excluded and the remaining six bins summed (Nolan et al., 2000).

### Genetic Mapping and Identification

Mice were raised by conventional mating (n = 6) and by in vitro fertilization (n = 83). DNA was extracted from all mice (n = 89) (Thornton et al., 1999). Polymorphic markers were amplified by PCR and assessed on 4% agarose gels stained with ethidium bromide. Results of the genome scan were analyzed using a regression analysis implemented in QTL cartographer (Basten et al., 1996). Known and predicted genes were identified using the *ensembl* gene database. All coding DNA was sequenced using a BigDye dideoxy-terminator system and analyzed on an ABI3700 sequencer (Applied Biosystems).

### Biochemical Experiments

A full-length cDNA encoding mouse *Tuba1* (Villasante et al., 1986) or a mutated form (S140G) generated by PCR and checked by DNA sequence analysis was subcloned into a pET23 vector (Novagen). These plasmids were used to express the corresponding unlabeled or ^35^S-methionine-labeled recombinant protein in host BL21(DE3) *E. coli* cells (Studier et al., 1990). Insoluble inclusion bodies were purified, unfolded in 7M urea (Gao et al., 1992), and quantitated by staining with Coomassie blue following resolution by SDS-PAGE. Unfolded, unlabeled wild-type or mutant Tuba1 probes (100ng) were used for in vitro folding assays (10 μl) containing the cytosolic chaperonin CCT, ATP, and α-^32^P-GTP (specific activity, 100 Ci/mmol) as described previously (Bartolini et al., 2002, Tian et al., 1999). In some in vitro folding experiments, and in those using ^35^S-methionine-labeled probes, folding reactions were supplemented with equimolar (with respect to CCT) amounts of the tubulin-specific chaperones B, C, D, and E and purified bovine brain tubulin. Reactions were incubated at 30°C for 1 hr and the products resolved by nondenaturing polyacrylamide gel electrophoresis (Gao et al., 1992). Transcription/translation reactions were performed in a cell-free translation cocktail (TnT® T7 Coupled Reticulocyte Lysate System, Promega) in the presence of ^35^S-methionine in accordance with the manufacturer's recommendations. Incubation was for 1 hr at 30°C. The reaction products were mixed with depolymerized brain microtubules (containing about 2 mg of total protein) and then taken through two cycles of polymerization/depolymerization. At the end of each of these cycles, an aliquot of the depolymerized material (representing one tenth of the total reaction mixture) was removed and analyzed by nondenaturing gel electrophoresis (Gao et al., 1992).

### Cellular Experiments

Wild-type and S140G mutant α-1 tubulin sequences were tagged at the C terminus by addition of sequences encoding the FLAG epitope and inserted into the pcDNA3.1(+) vector. C-terminal addition of small epitopes does not interfere with the normal incorporation of α-tubulin isotypes into microtubules (Gu and Cowan, 1989). Constructs were transfected into HeLa cells grown on glass coverslips in Dulbecco's Modified Eagle's Medium containing 10% foetal calf serum using the Fugene6 transfection reagent (Roche). Thirty-six hours posttransfection cells were fixed with cold methanol and stained with a rabbit polyclonal anti-β-tubulin antibody and a monoclonal anti-FLAG antibody (Sigma).

### BAC Rescue

BAC DNA was injected into the pronucleus of C3H/HeH embryos. Embryos were then transferred into the oviducts of 0.5 day pseudopregnant CD1 females and allowed to develop to term. Fifty-nine offspring were genotyped with a PCR-based assay to identify the BAC vector and flanking sequences. Mice carrying the transgene were crossed with *Jna/+* mice.

### Anatomical Studies

Eight-week-old mice were perfused with 0.9% NaCl and 4% paraformaldehyde, the brains removed and postfixed for 6 hr. Immunohistochemistry employed antibodies at the following concentrations: FOXP2 (Serotec;1:100), calbindin (Swant; 1:5000), Er81 (1:32000), Cux-1 (Santa Cruz; 1:100), Brn-1 (Santa Cruz; 1:200) and NeuN (Chemicon; 1:400). A biotinylated secondary antibody (Vector; 1:200) was used. Fluorescein isothiocyanate (FITC)-labeled streptavidin (Molecular Probes; 1:500) permitted visualization of staining with a TE-2000 inverted microscope (Nikon). Golgi staining was performed on 160 μm thick sections utilizing a Rapid GolgiStain Kit (FD Neurotechnologies). Scion Image software (NIH) was used to define a line from the center of the soma through the adjacent 100 μm of the apical dendrite (Demyanenko et al., 2004). Apical dendrites in all three neocortical regions were measured, and misorientated dendrites were defined as those with 1.5 standard deviations from the mean (θ > 8°). Inverted dendrites were defined as those where θ > 90°. Dendrites were assessed blind to the genotype.

### In Vivo Neuronal Migration Assay

Pregnant mice were injected intraperitoneally with BrdU at 50 μg/g of body weight at E12.5, E14.5, and E16.5. Brains from littermates were harvested at P0, drop fixed in 4% PFA, and then 14 μM sections cut on a cryostat. Antigen retrieval was performed as previously described (Huang and Herbert, 2005) before incubation with BrdU sera (Accurate Chemical & Scientific; 1:100) and detection with a biotinylated secondary antibody (Vector; 1:500). The cortex was divided into ten equal bins, extending from the intermediate zone to the cortical surface and BrdU-positive cells counted. Multiple sections (n = 4) were analyzed for each animal (n = 3–4) for each genotype. Cells were counted blind to the genotype. Comparisons between littermate mutant and wild-type animals were analyzed by a linear mixed model by restricted estimate maximum likelihood using the nlme package (Pinheiro and Bates, 2000) in R (R-Development-Core-Team, 2004).

### Behavioral Phenotyping

All behavioral phenotyping was performed on mice between the ages of 8 and 14 weeks. Open-field and elevated plus maze assessment was as previously described (Solberg et al., 2006). The apparatus for the nonspatial reference memory task and for spontaneous and rewarded alternation consisted of an enclosed T-maze constructed from dark gray painted wood. The dimensions of each arm were 30 cm × 10 cm × 29 cm. The nonspatial reference memory task utilized goal arm inserts (carpet, sandpaper, thin wire mesh, or wire bars). Control and mutant mice were then assigned an insert to match a food reward in a counterbalanced protocol. The position of the goal arm inserts (and therefore the food reward) varied in a pseudorandom fashion. Mice had to learn that a particular insert was associated with a food reward. Each mouse underwent ten trials a day, 20 min between trials, for 6 days. Mice that made the correct goal arm choice were permitted to consume the food reward. If not, they were allowed to explore the incorrect goal arm for 30 s and were then removed. To control for the possibility of odor cues, the food reward was only provided following arm choice on the final day. Spontaneous and rewarded alternation was assessed as previously described (Bannerman et al., 2004), except for rewarded alternation where a free choice on the sample run was permitted. Each mouse underwent 20 trials with an intertrial interval of approximately 20 min. Nesting, rotarod, and static rods assessments were performed as previously described (Deacon et al., 2002).

### Mutation Detection in Humans

PCR products were subjected to DHPLC by the WAVE nucleic-acid-fragment analysis system (Transgenomic). Patients with abnormal profiles were sequenced with the BigDye dideoxy-terminator system, analyzed on an ABI3700 sequencer (Applied Biosystems). Difference in the frequency of mutations between cases and controls was tested by chi-square statistic, with the P value obtained by simulation using the chi-square test in R (R-Development-Core-Team, 2004).

## Supplemental Data

Document S1. Four Figures

## References

[bib1] Angevine J.B. (1965). Time of neuron origin in the hippocampal region. An autoradiographic study in the mouse. Exp. Neurol..

[bib2] Bannerman D.M., Deacon R.M., Brady S., Bruce A., Sprengel R., Seeburg P.H., Rawlins J.N. (2004). A comparison of GluR-A-deficient and wild-type mice on a test battery assessing sensorimotor, affective, and cognitive behaviors. Behav. Neurosci..

[bib3] Bartolini F., Bhamidipati A., Thomas S., Schwahn U., Lewis S.A., Cowan N.J. (2002). Functional overlap between retinitis pigmentosa 2 protein and the tubulin-specific chaperone cofactor C. J. Biol. Chem..

[bib4] Basten C.J., Zeng Z.B., Weir B.S. (1996). QTLCartographer: a suite of programs for mapping quantitative trait loci. Abstracts to Plant Genome, IV.

[bib5] Boycott K.M., Flavelle S., Bureau A., Glass H.C., Fujiwara T.M., Wirrell E., Davey K., Chudley A.E., Scott J.N., McLeod D.R., Parboosingh J.S. (2005). Homozygous deletion of the very low density lipoprotein receptor gene causes autosomal recessive cerebellar hypoplasia with cerebral gyral simplification. Am. J. Hum. Genet..

[bib6] Buhot M.C., Dubayle D., Malleret G., Javerzat S., Segu L. (2001). Exploration, anxiety, and spatial memory in transgenic anophthalmic mice. Behav. Neurosci..

[bib7] Churchill G.A., Doerge R.W. (1994). Empirical threshold values for quantitative trait mapping. Genetics.

[bib8] Corbo J.C., Deuel T.A., Long J.M., LaPorte P., Tsai E., Wynshaw-Boris A., Walsh C.A. (2002). Doublecortin is required in mice for lamination of the hippocampus but not the neocortex. J. Neurosci..

[bib9] Deacon R.M., Croucher A., Rawlins J.N. (2002). Hippocampal cytotoxic lesion effects on species-typical behaviours in mice. Behav. Brain Res..

[bib10] Demyanenko G.P., Schachner M., Anton E., Schmid R., Feng G., Sanes J., Maness P.F. (2004). Close homolog of L1 modulates area-specific neuronal positioning and dendrite orientation in the cerebral cortex. Neuron.

[bib11] des Portes V., Pinard J.M., Billuart P., Vinet M.C., Koulakoff A., Carrie A., Gelot A., Dupuis E., Motte J., Berwald-Netter Y. (1998). A novel CNS gene required for neuronal migration and involved in X-linked subcortical laminar heterotopia and lissencephaly syndrome. Cell.

[bib12] Dobyns W.B., Truwit C.L. (1995). Lissencephaly and other malformations of cortical development: 1995 update. Neuropediatrics.

[bib13] Farr S.A., Banks W.A., La Scola M.E., Morley J.E. (2002). Blind mice are not impaired in T-maze footshock avoidance acquisition and retention. Physiol. Behav..

[bib14] Francis F., Meyer G., Fallet-Bianco C., Moreno S., Kappeler C., Socorro A.C., Tuy F.P., Beldjord C., Chelly J. (2006). Human disorders of cortical development: from past to present. Eur. J. Neurosci..

[bib15] Gao Y., Thomas J.O., Chow R.L., Lee G.H., Cowan N.J. (1992). A cytoplasmic chaperonin that catalyzes beta-actin folding. Cell.

[bib16] Gleeson J.G., Allen K.M., Fox J.W., Lamperti E.D., Berkovic S., Scheffer I., Cooper E.C., Dobyns W.B., Minnerath S.R., Ross M.E., Walsh C.A. (1998). Doublecortin, a brain-specific gene mutated in human X-linked lissencephaly and double cortex syndrome, encodes a putative signaling protein. Cell.

[bib17] Gloster A., El-Bizri H., Bamji S.X., Rogers D., Miller F.D. (1999). Early induction of Talpha1 alpha-tubulin transcription in neurons of the developing nervous system. J. Comp. Neurol..

[bib18] Gu W., Cowan N.J. (1989). Assembly properties of altered beta-tubulin polypeptides containing disrupted autoregulatory domains. Mol. Cell. Biol..

[bib19] Gupta A., Tsai L.H., Wynshaw-Boris A. (2002). Life is a journey: a genetic look at neocortical development. Nat. Rev. Genet..

[bib20] Hong S.E., Shugart Y.Y., Huang D.T., Shahwan S.A., Grant P.E., Hourihane J.O., Martin N.D., Walsh C.A. (2000). Autosomal recessive lissencephaly with cerebellar hypoplasia is associated with human RELN mutations. Nat. Genet..

[bib21] Huang G.J., Herbert J. (2005). Serotonin modulates the suppressive effects of corticosterone on proliferating progenitor cells in the dentate gyrus of the hippocampus in the adult rat. Neuropsychopharmacology.

[bib22] Kappeler C., Saillour Y., Baudoin J.P., Tuy F.P., Alvarez C., Houbron C., Gaspar P., Hamard G., Chelly J., Metin C., Francis F. (2006). Branching and nucleokinesis defects in migrating interneurons derived from doublecortin knockout mice. Hum. Mol. Genet..

[bib23] Kikkawa M., Hirokawa N. (2006). High-resolution cryo-EM maps show the nucleotide binding pocket of KIF1A in open and closed conformations. EMBO J..

[bib24] Leventer R.J. (2005). Genotype-phenotype correlation in lissencephaly and subcortical band heterotopia: the key questions answered. J. Child Neurol..

[bib25] Lewis S.A., Tian G., Vainberg I.E., Cowan N.J. (1996). Chaperonin-mediated folding of actin and tubulin. J. Cell Biol..

[bib26] Lowe J., Li H., Downing K.H., Nogales E. (2001). Refined structure of alpha beta-tubulin at 3.5 A resolution. J. Mol. Biol..

[bib27] Moores C.A., Perderiset M., Francis F., Chelly J., Houdusse A., Milligan R.A. (2004). Mechanism of microtubule stabilization by doublecortin. Mol. Cell.

[bib28] Nolan P.M., Peters J., Strivens M., Rogers D., Hagan J., Spurr N., Gray I.C., Vizor L., Brooker D., Whitehill E. (2000). A systematic, genome-wide, phenotype-driven mutagenesis programme for gene function studies in the mouse. Nat. Genet..

[bib29] Paylor R., Hirotsune S., Gambello M.J., Yuva-Paylor L., Crawley J.N., Wynshaw-Boris A. (1999). Impaired learning and motor behavior in heterozygous Pafah1b1 (Lis1) mutant mice. Learn. Mem..

[bib30] Phillips J.B., Lyczak R., Ellis G.C., Bowerman B. (2004). Roles for two partially redundant alpha-tubulins during mitosis in early Caenorhabditis elegans embryos. Cell Motil. Cytoskeleton.

[bib31] Pinheiro J.C., Bates D.M. (2000). Mixed effects models in S and S-PLUS.

[bib32] Qiu S., Korwek K.M., Pratt-Davis A.R., Peters M., Bergman M.Y., Weeber E.J. (2005). Cognitive disruption and altered hippocampus synaptic function in Reelin haploinsufficient mice. Neurobiol. Learn. Mem..

[bib33] R-Development-Core-Team (2004). A language and environment for statistical computing.

[bib34] Reiner O., Carrozzo R., Shen Y., Wehnert M., Faustinella F., Dobyns W.B., Caskey C.T., Ledbetter D.H. (1993). Isolation of a Miller-Dieker lissencephaly gene containing G protein beta-subunit-like repeats. Nature.

[bib35] Solberg L.C., Valdar W., Gauguier D., Nunez G., Taylor A., Burnett S., Arboledas-Hita C., Hernandez-Pliego P., Davidson S., Burns P. (2006). A protocol for high-throughput phenotyping, suitable for quantitative trait analysis in mice. Mamm. Genome.

[bib36] Spiegelman B.M., Penningroth S.M., Kirschner M.W. (1977). Turnover of tubulin and the N site GTP in Chinese hamster ovary cells. Cell.

[bib37] Studier F.W., Rosenberg A.H., Dunn J.J., Dubendorff J.W. (1990). Use of T7 RNA polymerase to direct expression of cloned genes. Methods Enzymol..

[bib38] Takahashi T., Goto T., Miyama S., Nowakowski R.S., Caviness V.S. (1999). Sequence of neuron origin and neocortical laminar fate: relation to cell cycle of origin in the developing murine cerebral wall. J. Neurosci..

[bib39] Thornton C.E., Brown S.D., Glenister P.H. (1999). Large numbers of mice established by in vitro fertilization with cryopreserved spermatozoa: implications and applications for genetic resource banks, mutagenesis screens, and mouse backcrosses. Mamm. Genome.

[bib40] Tian G., Vainberg I.E., Tap W.D., Lewis S.A., Cowan N.J. (1995). Quasi-native chaperonin-bound intermediates in facilitated protein folding. J. Biol. Chem..

[bib41] Tian G., Bhamidipati A., Cowan N.J., Lewis S.A. (1999). Tubulin folding cofactors as GTPase-activating proteins. GTP hydrolysis and the assembly of the alpha/beta-tubulin heterodimer. J. Biol. Chem..

[bib42] Tian G., Huang M.C., Parvari R., Diaz G.A., Cowan N.J. (2006). Cryptic out-of-frame translational initiation of TBCE rescues tubulin formation in compound heterozygous HRD. Proc. Natl. Acad. Sci. USA.

[bib43] Vega L.R., Fleming J., Solomon F. (1998). An alpha-tubulin mutant destabilizes the heterodimer: phenotypic consequences and interactions with tubulin-binding proteins. Mol. Biol. Cell.

[bib44] Villasante A., Wang D., Dobner P., Dolph P., Lewis S.A., Cowan N.J. (1986). Six mouse alpha-tubulin mRNAs encode five distinct isotypes: testis-specific expression of two sister genes. Mol. Cell. Biol..

[bib45] Willins D.A., Xiang X., Morris N.R. (1995). An alpha tubulin mutation suppresses nuclear migration mutations in Aspergillus nidulans. Genetics.

[bib46] Zeng Z.B. (1994). Precision mapping of quantitative trait loci. Genetics.

