# Supplementary material for: Mutations in α-Tubulin Cause Abnormal Neuronal Migration in Mice and Lissencephaly in Humans
Source: Cell. 2007 Jan 12;128(1):45–57. doi: 10.1016/j.cell.2006.12.017 (PMC1885944; doi:10.1016/j.cell.2006.12.017)
Supplement: Document S1. Four Figures [file mmc1.pdf]

Cell, Volume *128*
Supplemental Data

Mutations in -Tubulin Cause Abnormal Neuronal Migration in Mice and Lissencephaly in Humans

David A. Keays, Guoling Tian, Karine Poirier, Guo-Jen Huang, Christian Siebold, James Cleak,Peter L. Oliver, Martin Fray, Robert J Harvey, Zoltán Molnár, Maria C. Piñon, Neil Dear,William Valdar, Steve D. M. Brown, Kay E. Davies, J. Nicholas P. Rawlins, Nicholas J. Cowan, Patrick Nolan, Jamel Chelly, Jonathan Flint

Figure S1

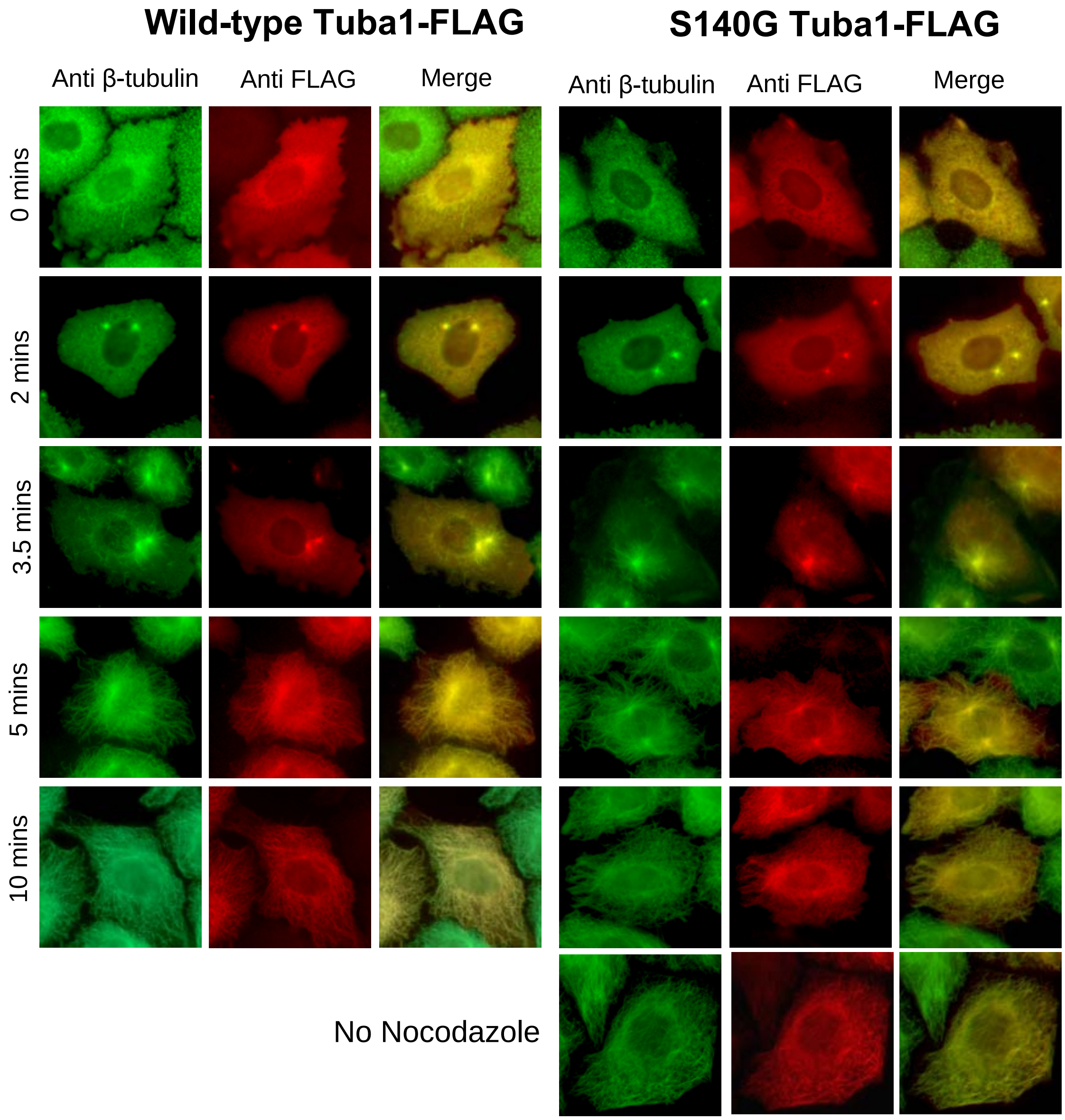

Figure S1: HeLa cells grown on glass coverslips were transfected with constructs encoding FLAG-tagged wild type or S140G mutant Tuba1 as described (see Methods). 36 hours post transfection, the cultures were treated for 2 hours with nocodazole (10 μM) to completely depolymerize microtubules. Cells were restored to warm (37ºC) drug-free medium and processed for immunofluorescence using anti-FLAG and anti-β-tubulin antisera at the times shown on the left of the Figure.

Figure S2

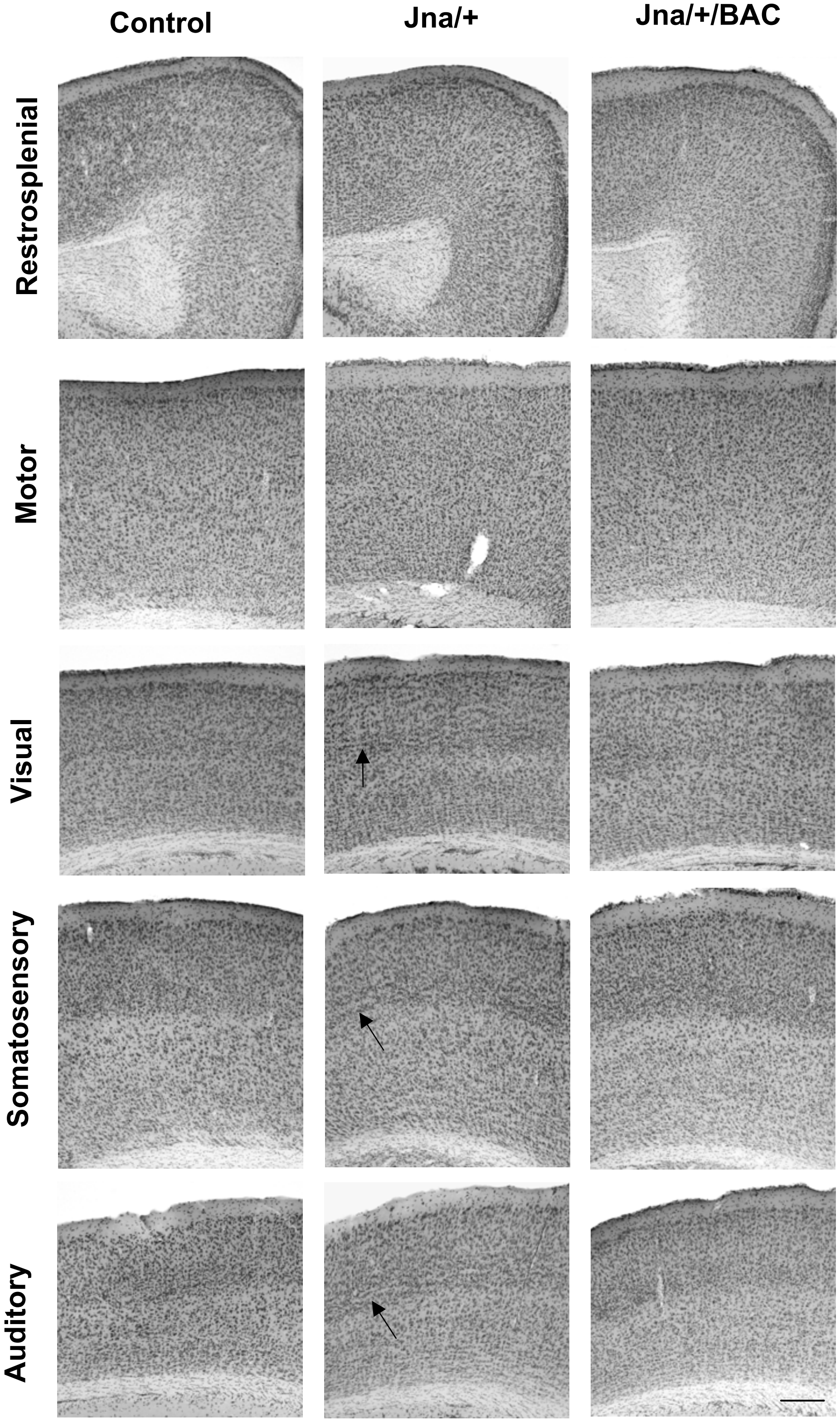

Figure S2: Nissl stains of the retrosplenial, motor, visual, somatosensory and auditory cortices. Arrows indicate wave-like perturbations in layer IV of the visual, somatosensory and auditory cortices. Scale bar shows 200 µm.

Figure S3

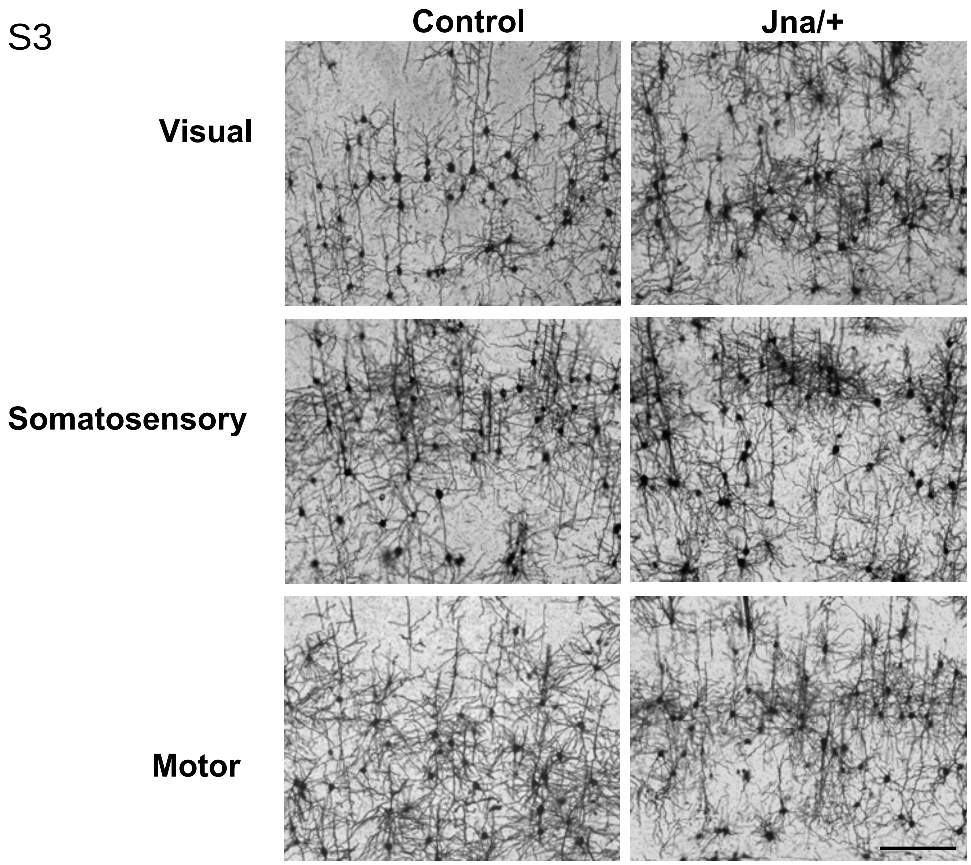

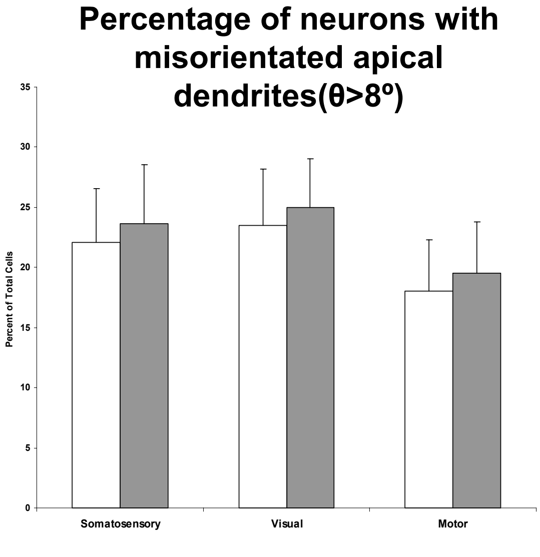

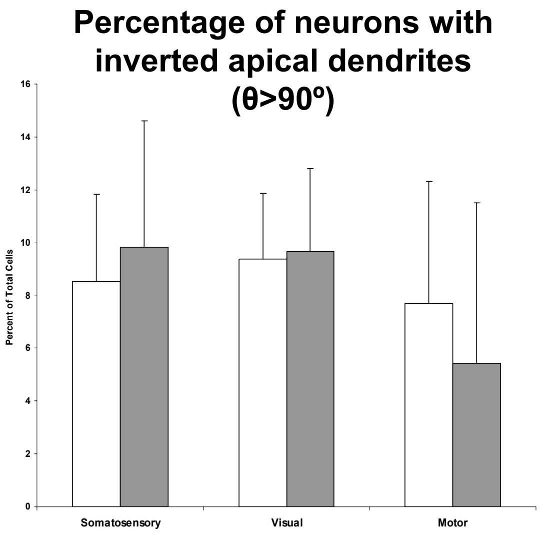

Figure S3: Dendritic orientation in the visual, somatosensory and motor cortices in littemate control (n=3) and *Jna*/+ mice (n=3). No differences were observed in the percentage of neurons with misorientated (θ>8º) apical dendrites or with inverted apical dendrites (θ>90º). Scale bar shows 200 µm.

Figure S4

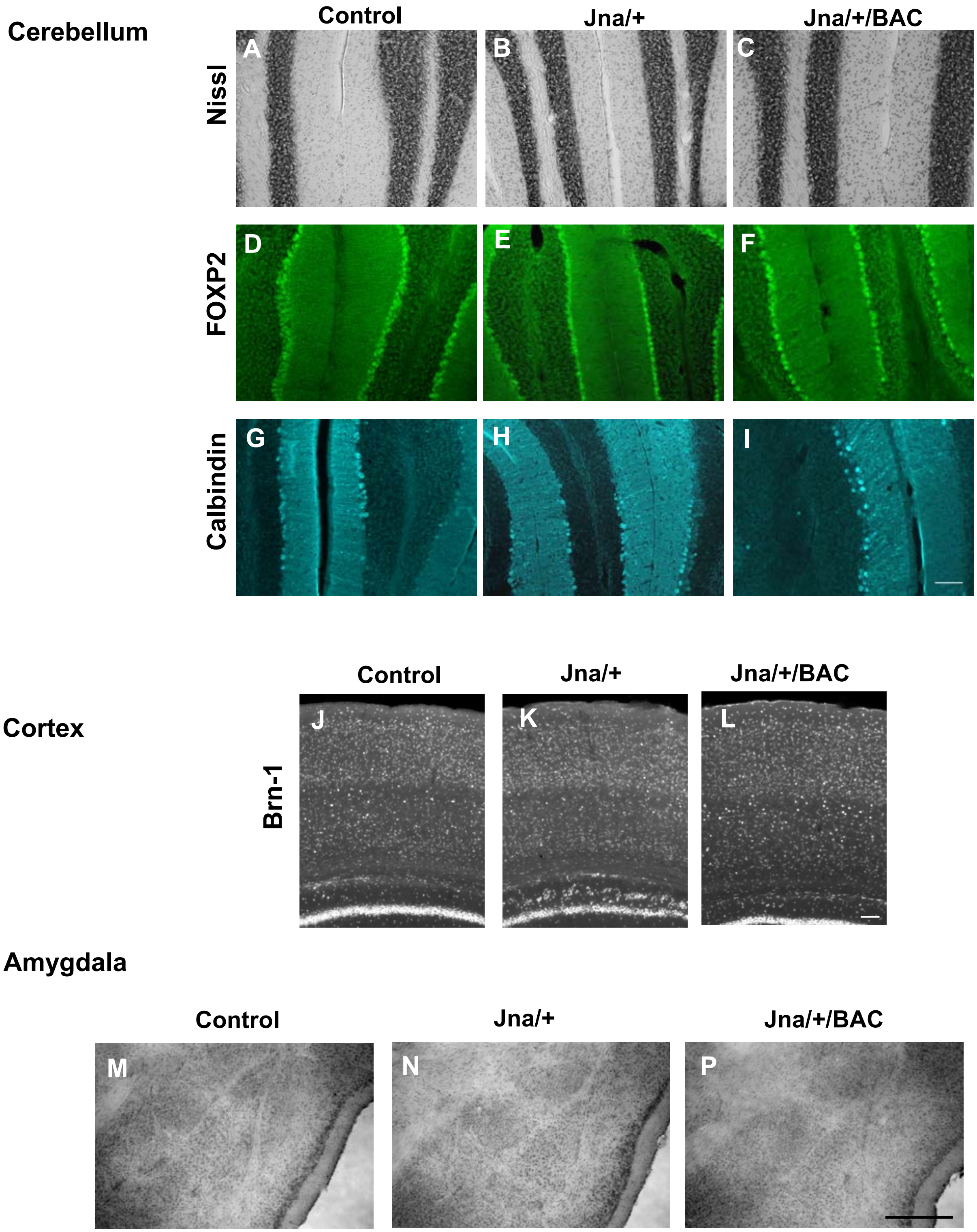

Figure S4: Panels A-I show cresyl violet, FOXP2 and calbindin staining of the cerebellum. No abnormalities in the laminar structure of the cerebellum were observed in Jna/+ mice . Scale bar shows 200 µm. Panels J-K show Brn-1 staining in the cortex, confirming an intact laminar structure. Scale bar shows 100 µm. Panels M to P show cresyl violet staining of the amygdala (M-P). The amygdala appears normal in mutant animals. Scale bar shows 500µm.
